# Feasibility of Goal-Directed Fluid Therapy in Patients with Transcatheter Aortic Valve Replacement - An Ambispective Analysis

**DOI:** 10.21470/1678-9741-2022-0470

**Published:** 2024-02-21

**Authors:** Ralf Felix Trauzeddel, Michael Nordine, Giovanni B. Fucini, Michael Sander, Henryk Dreger, Karl Stangl, Sascha Treskatsch, Marit Habicher

**Affiliations:** 1 Department of Anesthesiology and Intensive Care Medicine, Charité - Universitätsmedizin Berlin, Corporate Member of Freie Universität Berlin and Humboldt-Universität zu Berlin, Campus Benjamin Franklin, Berlin, Germany; 2 Department of Anesthesiology, Intensive Care Medicine, and Pain Therapy, University Hospital Frankfurt, Goethe University Frankfurt, Frankfurt, Hessen, Germany; 3 Institute of Hygiene and Environmental Medicine and National Reference Center for the Surveillance of Nosocomial Infections, Charité - Universitätsmedizin Berlin, Corporate Member of Freie Universität Berlin and Humboldt-Universität zu Berlin, Berlin, Germany; 4 Department of Anesthesiology, Operative Intensive Care Medicine, and Pain Therapy, Justus Liebig University of Giessen, Hessen, Germany; 5 Department of Cardiology, Angiology, and Intensive Care Medicine, Deutsches Herzzentrum der Charité - Medical Heart Center of Charité and German Heart Institute Berlin, Campus Virchow-Klinikum, Berlin, Germany; 6 Department of Cardiology and Angiology, Deutsches Herzzentrum der Charité - Medical Heart Center of Charité and German Heart Institute Berlin, Campus Charité Mitte, Berlin, Germany

**Keywords:** Atrial Fibrilation, Transcatheter Aortic Valve Replacemnet, Stroke Volume, Control Groups, Incidence, Cardiac Conduction System Disease, Fluid Therapy, Ischemia, Delirium

## Abstract

**Introduction:**

Goal-directed fluid therapy (GDFT) has been shown to reduce postoperative complications. The feasibility of GDFT in transcatheter aortic valve replacement (TAVR) patients under general anesthesia has not yet been demonstrated. We examined whether GDFT could be applied in patients undergoing TAVR in general anesthesia and its impact on outcomes.

**Methods:**

Forty consecutive TAVR patients in the prospective intervention group with GDFT were compared to 40 retrospective TAVR patients without GDFT. Inclusion criteria were age ≥ 18 years, elective TAVR in general anesthesia, no participation in another interventional study. Exclusion criteria were lack of ability to consent study participation, pregnant or nursing patients, emergency procedures, preinterventional decubitus, tissue and/or extremity ischemia, peripheral arterial occlusive disease grade IV, atrial fibrillation or other severe heart rhythm disorder, necessity of usage of intra-aortic balloon pump. Stroke volume and stroke volume variation were determined with uncalibrated pulse contour analysis and optimized according to a predefined algorithm using 250 ml of hydroxyethyl starch.

**Results:**

Stroke volume could be increased by applying GDFT. The intervention group received more colloids and fewer crystalloids than control group. Total volume replacement did not differ. The incidence of overall complications as well as intensive care unit and hospital length of stay were comparable between both groups. GDFT was associated with a reduced incidence of delirium. Duration of anesthesia was shorter in the intervention group. Duration of the interventional procedure did not differ.

**Conclusion:**

GDFT in the intervention group was associated with a reduced incidence of postinterventional delirium.

**Table:** 

Abbreviations, Acronyms & Symbols				
AKIN	= Acute Kidney Injury Network		LOS	= Length of stay
AS	= Aortic stenosis		LVEF	= Left ventricular ejection fraction
AUC_roc_	= Area under the receiver operating characteristic curve		MAC	= Monitored anesthesia care
BMI	= Body mass index		MAP	= Mean arterial pressure
CI	= Confidence interval		PACU	= Postanesthesia care unit
CO	= Cardiac output		POD	= Postoperative delirium
CVP	= Central venous pressure		PPV	= Pulse pressure variation
DO₂	= Delivery of oxygen		RBC	= Red blood cells
EuroSCORE	= European System for Cardiac Operative Risk Evaluation		RCTs	= Randomized controlled trials
FFP	= Fresh frozen plasma		SOP	= Standard operating procedure
GDFT	= Goal-directed fluid therapy		SV	= Stroke volume
HAES	= Hydroxyethylstarch		SV_od_	= Stroke volume measured via esophageal Doppler
HF	= Heart frequency		SV_vig_	= Stroke volume measured via FloTrac®
IBP	= Invasive blood pressure		SVV	= Stroke volume variation
ICU	= Intensive care unit		TAVR	= Transcatheter aortic valve replacement
LBBB	= Left bundle branch block			

## INTRODUCTION

Transcatheter aortic valve replacement (TAVR) has become an alternative treatment for symptomatic patients with severe aortic stenosis (AS) not eligible for surgical aortic valve replacement due to a high periprocedural risk or relevant comorbidities^[1,3]^. Nevertheless, TAVR is still associated with possible periinterventional complications such as cardiac arrhythmias, renal failure, or neurological dysfunctions^[4]^.

The main anesthesiological objectives besides choice of the optimal anesthesia technique for the individualized patient are to maintain hemodynamic stability and sufficient tissue perfusion and oxygenation during the procedure. Optimization of preload is of particular importance to increase left ventricular stroke volume (SV) and thus delivery of oxygen (DO2). This can potentially be achieved by applying the concept of goal-directed fluid therapy (GDFT). Several randomized controlled trials (RCTs) as well as meta-analyses have shown that GDFT is associated with fewer postoperative complications and shorter hospital stays in surgery^[5,8]^. In the clinical routine, it is also shown that GDFT is feasible and associated with a better outcome^[9]^. Its concept has been applied to various intensive care medicine as well as non-cardiac and cardiac surgical patients^[10,14]^.

However, as far as the authors are aware, there exist no data on the feasibility of GDFT based on SV optimization during TAVR. Therefore, we examined whether GDFT could be applied in patients undergoing TAVR in general anesthesia. Additionally, we examined whether GDFT in TAVR would have an impact on postoperative outcomes compared to fluid replacement based on clinical standard without GDFT.

## METHODS

### Study Population

Patients in the intervention group were originally consecutive participants in a two-arm pilot study in intraoperative thermal management using a noninvasive warming system in minimally invasive heart valve replacement. As GDFT was also applied in the study, data were also analyzed regarding hemodynamic optimization in TAVR. Therefore, in this ambispective substudy, patients in the prospective intervention group with GDFT were compared with a retrospective control group before the hemodynamic optimization protocol was implemented. Inclusion criteria were age ≥ 18 years, elective TAVR in general anesthesia, and no participation in another interventional study. Exclusion criteria were lack of ability to consent study participation, pregnant or nursing patients, emergency procedures, preinterventional decubitus, tissue and/or extremity ischemia, peripheral arterial occlusive disease grade IV, atrial fibrillation or other severe heart rhythm disorders which impeded usage of uncalibrated pulse contour analysis because of its insufficient validity in these disorders, and necessity of usage of intra-aortic balloon pump. All procedures performed in studies involving humans were in accordance with the ethical standards of the institutional and/or national research committee and with the 1964 Declaration of Helsinki and its later amendments or comparable ethical standards. The study was approved by the local ethics committee at Charité - Universitätsmedizin Berlin (EA 1/142/10) and registered at ClinicalTrials.gov (NCT01176110). Informed written consent was obtained from all study patients in the intervention group. Data from patients in the retrospective control group before GDFT implementation were collected anonymously, therefore informed written consent was waived. The study was performed at the Charité - Universitätsmedizin Berlin, Campus Charité Mitte. Our study adheres to CONSORT guidelines.

### Study Protocol

General anesthesia during TAVR was induced according to our local standard operating procedure (SOP) with fentanyl (1-4 µg/kg^-1^) or remifentanil (0.5 µg/kg/min), etomidate (0.2 mg/kg), and cis-atracurium (0.1 mg/kg), if necessary. Anesthesia was maintained with a continuous infusion of propofol (4-6 mg/kg^-1^ h^-1^) and remifentanil (0.1-0.2 µg/kg^-1^ min^-1^). Lungs of patients were ventilated with pressure control ventilation with a tidal volume of 8-10 ml/kg^-1^ ideal body weight. End-tidal CO₂ was kept between 35 and 40 mmHg. Before induction of general anesthesia, hemodynamic monitoring was established including invasive blood pressure measurement via right or left radial artery besides electrocardiogram, pulse oximetry, temperature, central venous pressure (CVP), and body core temperature through a urine catheter. Patients were extubated immediately after TAVR and transferred to an intensive care unit (ICU) or postanesthesia care unit (PACU) for further treatment and monitoring.

Haemodynamic optimization in the intervention group was performed based on SV monitored using a pulse contour method (Vigileo®, Edwards Lifesciences, Irvine, California, United States of America) and a special pressure transducer (FloTrac system®, Edwards Lifesciences). After determining individual baseline SV, an intravenous bolus of 250 ml of a colloid fluid replacement solution (6% hydroxyethylstarch [HAES] 130/0.4, Volulyte 6%®, Fresenius Kabi GmbH, Bad Homburg, Germany) was given within five minutes and consecutively repeated until no further increase of SV ≥ 10% could be achieved. The last successful fluid challenge resulting in an SV increase < 10% defined the optimum SV. In case of intraoperative decrease of SV, further fluid replacement was performed. After valve implantation, the optimal SV again was defined by infusion of 250 ml colloid. Responders (∆SV > 10%) received additional volume boluses until ∆SV was < 10%.

**Fig. 1:**
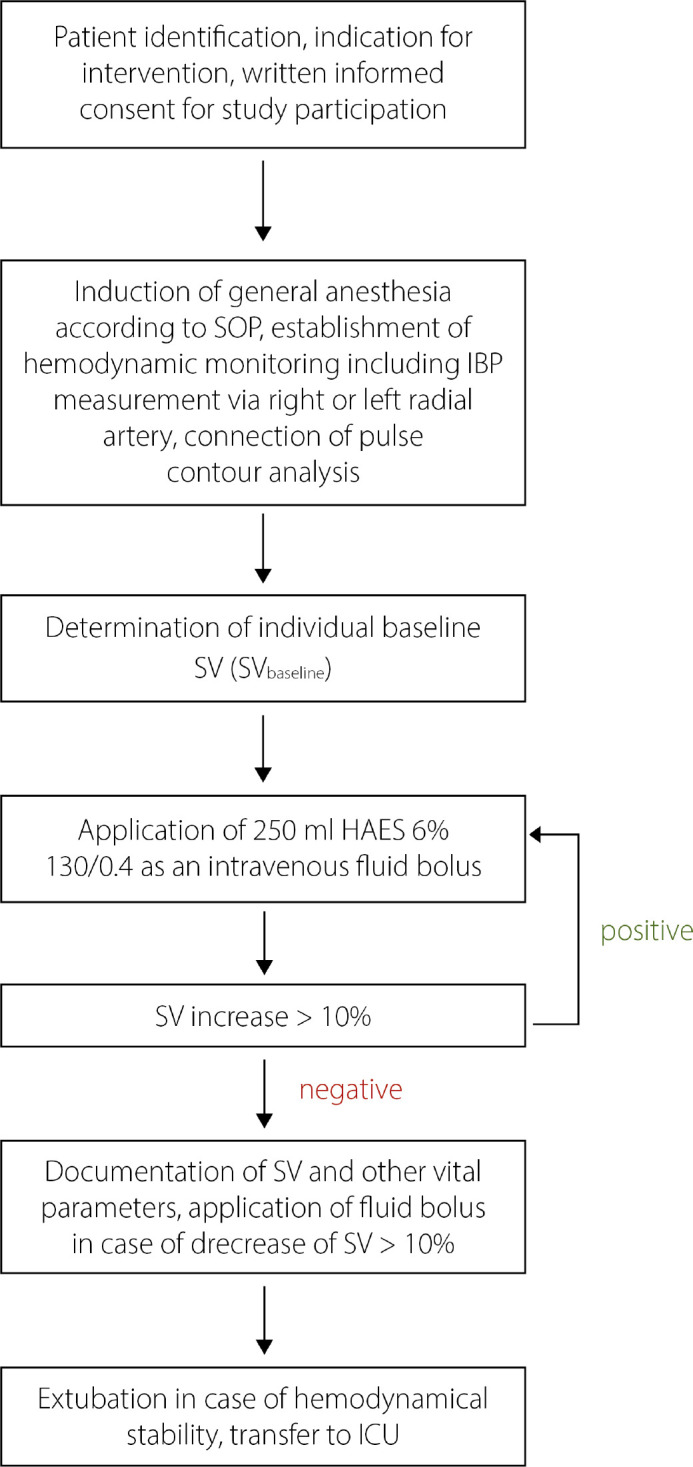
Representation of the study protocol. HAES=hydroxyethylstarch; IBP=invasive blood pressure; ICU=intensive care unit; SOP=standard operating procedure; SV=stroke volume.

Vasoactive medications were applied to maintain normotensive blood pressure values (mean arterial pressure [MAP] 65-100 mmHg, systolic blood pressure > 100 mmHg and < 140 mmHg). Inotropes were applied in case of insufficient increase in SV after fluid bolus according to the internal SOP of the department. Patients in the control group were monitored and treated at the discretion of the attending anesthesiologist based on internal SOP and clinical standard but without a fluid optimization strategy. The study protocol is represented in Figure 1.

### Outcome Variables

As the primary endpoint for the first study was the intravesical temperature at the end of the intervention, no explicit endpoint was defined for the current GDFT study. Regarding the feasibility of SV optimization by GDFT as the primary research question of this study, it was assumed that a case number of 40 study participants would be sufficient based on previous studies^[15]^. Other outcome variables were an increase of cardiac output (CO) and changes in SV variation (SVV) and complications, defined as delirium, infections (pneumoniae, urinary tract infection, wound infection), postoperative bleeding, acute kidney injury, cardiac or pulmonary complications or death of any cause as well as total length of hospital and ICU or PACU stay after TAVR, reduction in need of catecholamines or blood transfusions, reduction in postinterventional morbidity and mortality, duration of mechanically invasive ventilation, or dialysis.

### Statistical Analysis

Based on the previous pilot study character, a distinct power analysis was not performed, and an explorative data analysis was performed. Statistical analysis was done using IBM SPSS Statistics for Windows, version 21.0, Armonk, NY: IBM Corp. All data were checked for normal distribution using the Kolmogorov-Smirnov test. Non-normally distributed data are expressed as median with 25^th^ to 75^th^ percentiles, normally distributed data are expressed as mean and standard deviation. Morphometric and demographic data of both groups were examined for comparability by Mann-Whitney U test for non-normally distributed variables and Student’s *t*-test for unrelated samples for normally distributed variables. To evaluate the success of our intervention protocol, SV and SVV at different points in time of the intervention were compared using the Mann-Whitney U test. The occurrence of at least one postoperative complication was tested for independence using Fisher’s exact test for nominal variables. To test for statistical difference between both groups, primary and secondary outcome variables were compared using the Mann-Whitney U test, and the Fisher’s exact test was used for nominal variables.

## RESULTS

### Patients’ Characteristics

All study participants were treated between February 2010 and March 2011 at a single institution of the Charité - Universitätsmedizin Berlin (Berlin, Germany). Eighty patients undergoing elective TAVR were included: a) intervention group (N=40), and b) control group (N=40). Basic characteristics are shown in Table 1. There were no statistically significant differences between both groups in the demographic baseline data. The preoperative risk profile of the two patient groups according to the European System for Cardiac Operative Risk Evaluation (EuroSCORE) I, EuroSCORE II, and preoperative left ventricular ejection fraction also showed no significant differences. Transfemoral access was the predominant route in both groups.

**Table 1:** Baseline characteristics of the study population.

	Control group (n=40)	Interventional group (n=40)	*P*-value
Age (years)	83 (80;85)	81.5 (73;86)	0.3
Sex (female/male)	22 (55%)/18 (45%)	18 (45%)/22 (55%)	0.5
Body height (cm)	165.5 (158;174)	168 (160;175)	0.5
Body weight (kg)	69.5 (58;78)	74.5 (66;88)	0.053
BMI (kg/m^2^)	24.4 (23;27.5)	26.8 (23.6;30.3)	0.14
LVEF (%)	50 (43;60)	58 (45;60)	0.37
Access site			0.38
Transfemoral	31 (77.5%)	35 (87.5%)	
Transapical	9 (22.5%)	5 (12.5%)	
EuroSCORE I	17.8 (9.8;30)	15 (5.7;21.7)	0.3
EuroSCORE II	5.7 (3.7;11.6)	4.3 (2.7;8.3)	0.86

Parameters are shown as median and (25^th^ percentile;75^th^ percentile)

BMI=body mass index; EuroSCORE=European System for Cardiac Operative Risk Evaluation; LVEF=left ventricular ejection fraction

### Outcome Parameters

**Fig. 2:**
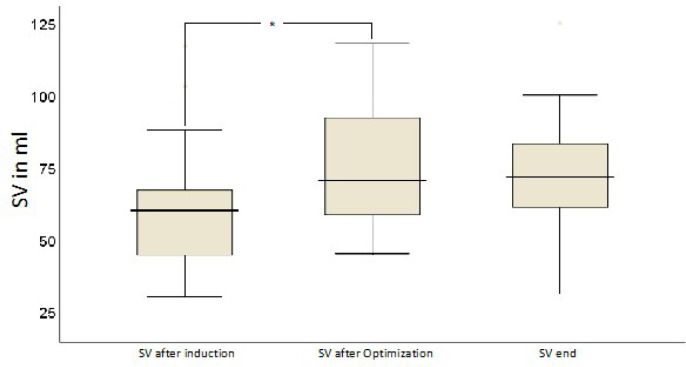
Responses of stroke volume (SV) to fluid boluses. The * indicates a positive increase in SV (central illustration).

**Fig. 3:**
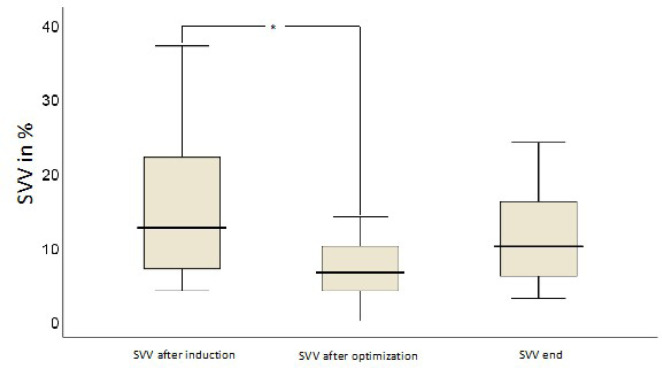
Responses of stroke volume variation (SVV) to fluid boluses. The * indicates a decrease in SVV.

The course of hemodynamic parameters during TAVR in the interventional group can be seen in Table 2. After induction of general anesthesia, there was a decrease in the MAP and heart frequency (*P*<0.01). GDFT resulted in an increase in SV (*P*=0.003), MAP (*P*=0.003), CVP (*P*=0.01), and CO (*P*=0.003) as well as a decrease in SVV (*P*=0.01) after first fluid optimization. SV remained elevated until the end of the intervention (Figure 2). In contrast, SVV was not lower at the end compared to after the first optimization (Figure 3). On average, in median, two (1;2.75) fluid boluses were necessary for optimization after induction, compared to one (1;2) after implantation of the aortic valve. More colloid and fewer crystalloid solutions were given in the intervention group than in the control group. Total volume replacement as well as total amount of blood products substituted were comparable as well as maximum dosage of norepinephrine intraoperatively and cumulative dosage of norepinephrine during intensive care (Table 3).

**Table 2:** Course of hemodynamic parameters during transcatheter aortic valve replacement in the interventional group.

	After induction	After 1^st^ optimization	After valve implantation	After 2^nd^ optimization	End of intervention
MAP	71 (60;83.2)	82 (72;91)	74 (65;81)	73 (65;81)	73 (66;80)
HF	66 (58;72)	63 (59;73)	71 (63;78)	68 (9.2)	68 (60;76)
CVP	9 (8;13.5)	15 (10;17)	15 (12;17)	15 (11;16)	13.5 (10;15)
CO	3.8 (3;4.7)	4.7 (3.8;5.6)	4.7 (4.1;5.9)	5 (4.1;5.9)	5 (3.9;5.6)
SV	60 (42;67)	70.5 (58;92.5)^*^	10.5 (5;18.2)	74 (64.2;87.5)	71.5 (61;8)
SVV	13 (7.7;22)	8 (4.2;13.7)^*^	10.5 (5;18.2)	9.5 (6;14)	10 (6;17.5)

Parameters are shown as median (25^th^ percentile;75^th^ percentile)

^*^Positive increase in SV

CO=cardiac output; CVP=central venous pressure; HF=heart frequency; MAP=mean arterial pressure; SV=stroke volume; SVV=stroke volume variation

**Table 3:** Intrainterventional volume replacement and catecholamine dosage.

	Control group (n=40)	Interventional group (n=40)	*P*-value
Colloids (ml)	500 (0;500)	750 (500;1000)	< 0.001
Crystalloids (ml)	500 (500;1000)	500 (0;500)	< 0.001
RBC (ml)	0 (0;300)	0 (0;150)	0.91
FFP (ml)	0 (0;0)	0 (0;0)	0.84
Total volume replacement (ml)	1000 (1000;1600)	1250 (1000;1500)	0.3
Maximum intraoperative norepinephrine dosage (µg/kg/min.)	0.05 (0.02;0.09)	0.05 (0.03;0.07)	0.67
Cumulative norepinephrine dosage on ICU (µg)	0.0 (0.0;0.0)	0.0 (0.0;0.15)	0.9

Parameters are shown as median (25^th^ percentile;75^th^ percentile)

FFP=fresh frozen plasma; ICU=intensive care unit; RBC=red blood cells

Duration of anesthesia was shorter in the intervention group, whereas duration of the interventional procedure was not different. There were no differences regarding ICU and hospital length of stay (LOS) and duration of invasive mechanical ventilation (Table 4).

**Table 4:** Intraand postinterventional patient characteristics.

	Control group (n=40)	Interventional group (n=40)	*P*-value
Length of anesthesia (min.)	148 (121;170)	120 (91;153)	0.003
Length of intervention (min.)	83 (70;93)	70 (60;94)	0.09
Length of hospital stay (days)	11 (7;14)	8 (7;16)	0.79
Length of ICU stay (days)	3 (2;6)	2 (1;6)	0.16
Need for post-procedural mechanical ventilation (n)	16	16	
Length of postinterventional mechanical ventilation (min.)	0 (0;55)	0 (0;255)	0.54

Parameters are shown as median (25^th^ percentile;75^th^ percentile)

ICU=intensive care unit

Thirty-one GDFT patients (77.5%) and 34 control patients (85%) suffered from at least one of the abovementioned complications. The number of complications per patient did not differ between groups (1.5 [1;3.5] *vs.* 2 [1;4], intervention group and control group, respectively). However, GDFT was associated with reduced rate of delirium (risk ratio 0.24; 95% confidence interval [CI] 0.08;0.7). See Table 5 for mortality and complications.

**Table 5:** Rate of complications.

	Control group	Interventional group	*P*-value
	(n=40)	(n=40)	
Total mortality	3 (7.5%)	3 (7.5%)	1
Delirium	17 (42.5%)	6 (15%)	0.006
Infectious complications	16 (40%)	22 (55%)	
Pneumoniae	9 (22.5%)	12 (30%)	0.61
Urinary tract infections	4 (10%)	2 (5%)	0.68
Others/unclear	4 (10%)	9 (22,5%)	0.13
Bleeding complications	9 (22.5%)	15 (37.5%)	0.22
Cardiovascular complications	16 (40%)	22 (55%)	
LBBB	9 (22.5%)	9 (22.5%)	1
Atrioventricular block (2^nd^-3^rd^ degrees)	2 (5%)	8 (20%)	0.09
Absolute arrhythmia	5 (12.5%)	3 (7.5%)	0.71
Stroke	1 (2.5%)	1 (2.5%)	1
Others	0	1 (2.5%)	
Pulmonary complications	10 (25%)	13 (32.5%)	0.62
Acute kidney failure	8 (20%)	6 (15%)	0.77

Parameters are shown as absolute values and percentages

LBBB=left bundle branch block

## DISCUSSION

In this study, GDFT with colloids has been shown to be able to optimize SV amongst patients undergoing TAVR in general anesthesia. As per protocol, patients in the GDFT group received more colloid infusions and fewer crystalloid infusions than those in the control group. The total administered volume between both groups, however, did not differ. Time spent under anesthesia in the GDFT group was shorter. Though this study was not powered for, SV optimization and shorter anesthesia duration were associated with a lower incidence of post-interventional delirium.

The optimization of perioperative DO₂ to the organs through administration of targeted volume boluses during cardiac and non-cardiac surgery has previously been described and successfully implemented into clinical routine^[9,16,17]^. We thus aimed to examine the translation of this effective intraoperative strategy for the first time during TAVR. In our protocol, SV was optimized using colloid solution immediately following induction of anesthesia. On average, in median, the protocol-driven administration of two (1;2.75) fluid boluses was sufficient to optimize SV, which may be interpreted as the absence of hemodynamic relevant fluid shift during TAVR. Interestingly, yet a significant decrease from baseline value after initial fluid challenge was detected for SVV, SVV did not remain lower at the end compared to after the first optimization in the course of the operation.

The use of pulse contour analysis amongst patients with high-grade AS has not been thoroughly examined. Certain validation studies involving surgical aortic valve replacement have shown non-optimal agreement between measured CO values via pulse contour analysis and thermodilution analysis, with a recommendation to measure trends rather than the absolute values^[18,20]^. Høiseth et al.^[21]^ examined 32 patients with high-grade AS and administered a 750 ml HAES bolus while measuring SV, SVV, and pulse pressure variation (PPV) via Flo Trac/Vigileo® monitoring during the preoperative period. The fluid challenge was repeated postoperatively on the ICU, and the same values were measured via esophageal Doppler. “Responders” were classified as showing a > 15% increase in SV after fluid challenge. A moderate predictive value for SVV and PPV preoperatively was shown (area under the receiver operating characteristic curve [AUC_roc_] 0.77 and 0.75). However, after aortic valve replacement the positive predictive value was improved (AUC_roc_ 0.90 and 0.95). The difference between the absolute value of the SV measured via esophageal Doppler (SV_od_) and via FloTrac® (SV_vig_) was high. Nevertheless, there was a good correlation between the change of SV_od_ and SV_vig_ before and after fluid challenge (trending ability). The authors thus concluded that the FloTrac® system can be used to monitor volume responsiveness amongst patients with high-grade AS^[22]^, which may be confirmed by our results. Petzoldt et al.^[20]^ showed that calibrated pulse contour analysis is valid and that in uncalibrated pulse contour measurements, the relative SV trend to be superior to single absolute values in 18 patients undergoing TAVR in severe AS. The dependency of the pulse contour analysis with the quality of the pulse curve is, however, an important limitation of the method. The high pressure gradient of the AS can alter the form of the pressure curve^[23]^ and could influence the measured value. Furthermore, the altered compliance of the left ventricle, as a consequence of left-sided myocardial hypertrophy/stiffness, can lead to a diastolic dysfunction. This may decrease the ability of the left ventricle to adequately respond to an increase in preload with an associated rise in SV^[24]^.

In this study, GDFT commenced immediately after anesthesia induction and prior to the start of the TAVR intervention. Due to the standardized fasting period, certain patients may have been hypovolemic before anesthesia induction. This may be further pronounced by the onset of anesthesia, which produces a relative hypovolemia^[25]^. Various other studies have concluded that a preoperative substitution with a crystalloid infusion can augment hepatic perfusion, but not necessarily renal perfusion^[27]^. Other groups have suggested that a preoperative crystalloid substitution offers no benefit for the patient^[28]^. In these studies, CO was examined only in the observation by Raue et al.^[26]^. They found that standard monitoring in awake patients offered no reliable information regarding the ideal timing or ideal amount of volume substitution needed. For this reason, the German Society of Anaesthesiology and Intensive Care Medicine, according to their S3 guidelines, has given the preoperative volume substitution an evidence rating of “Grade-B” (“can be given”), in order to replace an assumed volume deficit preoperatively, although no concrete evidence supports this recommendation. By individually optimizing SV, however, a targeted attempt has been shown to increase preload after induction of anesthesia in the here presented study.

As colloids were used in our protocol, it is obvious that only the intervention group received them in a larger amount. These results mirror that of other GDFT studies with similar protocols^[29,33]^. RCTs could show that there is, however, a potential nephrotoxic effect of colloid solutions and that the administration of hydroxyethyl starch to critically ill patients can have negative consequences^[34,36]^. The results of these RCTs led to restriction of use for colloid solutions in 2013, and ultimately to a suspension of approval from the Pharmacovigilance Risk Assessment Committee (or PRAC) in 2018. According to the S3 guidelines from the German Society of Anesthesiology and Intensive Care Medicine, critically ill patients with recently occurring coagulation or renal disorders should not be administered colloid solutions^[37]^. Our study took place before these restrictions and were in line with a consensus stating that colloid solutions can be used for hypovolemia and hemodynamic optimization amongst cardiosurgical patients^[37]^. Though this study was not powered for, no evidence of renal or other complications associated with SV optimization using colloids were observed. ICU and hospital LOS between the examined groups did not differ as well. Periand post-procedural bleeding occurred relatively frequently in both groups (37.5% GDFT *vs.* 22.5% control). This could be due to the transfemoral insertion method, as it has been previously described that this method is associated with a higher risk for vascular complications and hemorrhage compared to the transapical method (8-28% *vs.* 3.6-7%)^[38]^. Genereux et al.^[39]^ described the prevalence of bleeding and vascular complications to be 22.3% and 11.9%, respectively, and concluded that these complications have been underreported due to non-standardized definitions. They further noted an incidence of acute kidney injury (Acute Kidney Injury Network [AKIN] I-III) between 6.5% to 34.1% (pooled estimate rate 20.4%, 95% CI 16.2% to 25.8%), whereby the most cases (up to 26%) involve a light form of AKIN I[39]. In our study, a two-times increase of the preoperative creatinine was defined as renal failure, which equates to AKIN II. Therefore, according to our reporting, the total incidence of acute kidney injury was possibly underestimated by 20%.

In our study, 15% of the GDFT patients and 42.5% of the control patients developed postoperative delirium (POD). Information regarding the absolute incidence of POD for patients undergoing TAVR is still lacking in recent literature. Tse et al.^[40]^ found that the prevalence of POD in conventional coronary artery bypass grafting, surgical valve replacement, and TAVR is 28% in a retrospective analysis of 679 cases of POD. In a subgroup analysis of 122 post TAVR patients, a POD incidence of 27% was found, and patients undergoing TAVR with the transapical method showed significantly higher rates of POD compared with the transfemoral method (12% *vs.* 53%)^[41]^. Concerning our study, transapical and transfemoral access were utilized in equal ratios in both GDFT and control groups, so the cause of POD solely due to the implantation route may be neglected.

As reported, the duration of anesthesia in the GDFT group was shorter than in the control group. As the GDFT group underwent TAVR at a later time period than the control group, this difference could be due to a “learning effect”^[42]^. This experience has also been documented in another study^[43]^. However, there is evidence suggesting that exposition to deep^[44]^ and long-period sedation^[45]^ amongst intensive care patients is correlated with longer ventilation and hospital admission times, as well as increasing overall mortality. Additionally, other working groups have pointed out that deep sedation during ICU admission is a positive-predictive factor for the development of delirium^[46,47]^. The anti-cholinergic effect of many sedative agents has been described as a contributing factor of cerebral damage^[48]^. The exact cause is not clear at this time, and is most likely due to interactions with multiple central nervous system neuro-molecular pathways^[48,50]^. In conclusion, although our sample size was relatively small, there is evidence to suggest that GDFT and a shorter anesthesia time may be protective against the development of POD amongst patients undergoing TAVR. The exact cause of this remains unclear, however, GDFT can optimize cerebral perfusion and DO2, thereby reducing the degree of cerebral damage, and the shorter anesthesia time leads to shorter exposition time under anesthetic agents^[51]^. As stated before, more studies examining the role GDFT plays in improving POD are needed, as the financial and social costs of POD are immense.

### Limitations

This study has several limitations. First of all, this study was performed nearly 10 years ago. Nevertheless, it still demonstrates that SV can be optimized in TAVR patients. Secondly, in today’s clinical practice, a huge number of TAVR is performed under monitored anesthesia care (MAC). There are ambiguous results regarding outcome difference between MAC and general anesthesia^[52,53]^.

If additional GDFT in TAVR patients under MAC will be of any benefit, it must be evaluated in future studies. Third, this pilot study was a single-center analysis with a prospective intervention group and a retrospective control group. This ambispective study design by itself has intrinsic limitations. It cannot be ruled out that results are influenced by shorter duration of TAVR procedure and higher level of implantation skill of the team in the interventional group with increasing learning curve over time. Blinding for the intervention group was not planned or possible. The number of patients was not powered for any endpoint. Additionally, the follow-up was limited to hospital admission time. We did not registered preinterventional cerebrovascular function, SV, as well as aortic valve function. Additionally, we did not monitor urine output during the intervention. GDFT and targeted SV optimization are promising strategies for the anesthesiologist to improve perioperative outcomes amongst patients undergoing mid to high-risk surgeries. However, GDFT is not thoroughly studied amongst minimally invasive, although high-risk, procedures, such as TAVR. Uncalibrated pulse contour analysis technique might have been not the best choice for patients undergoing interventional heart valve procedures as these are based on nomograms of a healthy cohort. We could show that GDFT was possible amongst the intervention group, and that an optimization of SV using colloid-based fluid challenges is feasible. Other outcomes, being that of POD and anesthesia time, are not highly powered enough to draw a broader conclusion. Moreover, a lesser rate of POD might have been caused by shorter anesthesia and intervention time. Additionally, factors like frailty, which certainly contributes significantly to the prevalence of periinterventional POD, were not examined systematically in our study. RCTs with these outcomes in mind, with a high patient cohort, and longer follow-up times are needed in order to truly gauge the effectiveness of this strategy for broader use.

## CONCLUSION

In conclusion, to our knowledge, our study is the first attempt to apply GDFT to TAVR. Our protocol was feasible in optimizing SV. We noted a reduction in delirium but not in overall complications, overall mortality, and hospital and ICU LOS. Further studies are needed to show if this approach could achieve a better outcome for TAVR.
